# An improved approach for accurate and efficient calling of structural variations with low-coverage sequence data

**DOI:** 10.1186/1471-2105-13-S6-S6

**Published:** 2012-04-19

**Authors:** Jin Zhang, Jiayin Wang, Yufeng Wu

**Affiliations:** 1 Department of Computer Science and Engineering, University of Connecticut, Storrs, CT 06269, USA

## Abstract

**Background:**

Recent advances in sequencing technologies make it possible to comprehensively study structural variations (SVs) using sequence data of large-scale populations. Currently, more efforts have been taken to develop methods that call SVs with exact breakpoints. Among these approaches, split-read mapping methods can be applied on low-coverage sequence data. With increasing amount of data generated, more efficient split-read mapping methods are still needed. Also, since sequence errors can not be avoided for the current sequencing technologies, more accurate split-read mapping methods are still needed to better handle sequence errors.

**Results:**

In this paper, we present a split-read mapping method implemented in the program *SVseq2 *which improves our previous work SVseq1. Similar to SVseq1, SVseq2 calls deletions (and insertions) with exact breakpoints. SVseq2 achieves more accurate calling through split-read mapping within focal regions. SVseq2 also has a much desired feature: there is no need to specify the maximum deletion size, while some existing split-read mapping methods need more memory and longer running time when larger maximum deletion size is chosen. SVseq2 is also much faster because it only needs to examine a small number of ways of splitting the reads. Moreover, SVseq2 supports insertion calling from low-coverage sequence data, while SVseq1 only supports deletion finding. The program SVseq2 can be downloaded at http://www.engr.uconn.edu/~jiz08001/.

**Conclusions:**

SVseq2 enables accurate and efficient SV calling through split-read mapping within focal regions using paired-end reads. For many simulated data and real sequence data, SVseq2 outperforms some other existing approaches in accuracy and efficiency, especially when sequence coverage is low.

## Background

Finding structural genomic variations (e.g. deletions and insertions) has become an active research subject recently. It is commonly believed that some structural variations may be linked to complex diseases [1]. Now high throughput sequencing (HTS) technologies (such as the Roche 454 FLX, Illumina Genome Analyzer, and ABI SOLiD) become more available. Sequence data can potentially reveal nearly all genetic variations, including structural variants. Thus, great efforts have been made for discovering structural variations in populations using sequence data. For example, the ongoing 1000 Genomes Project has released called structural variations for several human populations from hundreds of sequenced individuals in the pilot studies [1].

Many current sequence datasets are consisted of pairs of reads. These pairs can be mapped to a reference genome using read mapping tools such as Bowtie [2] and BWA [3]. Usually both reads of the same pair can be successfully mapped to two different locations of the reference genome. The distance in between is called insert size, whose value depends on the library mean and standard deviation. Abnormal insert size (as suggested by the two mapped reads) may indicate the presence of some genomic structure not present in the reference genome. Such pairs are called discordant pairs, which can be useful in locating structural variations. There are many methods that detect SVs by analyzing the insert size of discordant pairs, such as PEMer [4], BreakDancer [5], GASV [6] and VariationHunter [7]. A drawback of these methods is that only approximate positions of the breakpoints of the SVs can be found, while the high resolution of break points is useful in SV classification and annotation [8]. Read depth methods (e.g. [9]) belong to another type of method that does not show the exact breakpoints.

Assembly and split-read mapping methods are the alternative approaches that can find exact breakpoints of SVs. One representative method using split-read mapping is the program Pindel [10]. Sometimes a reads mapping program cannot properly map a pair of reads. There are multiple causes for unmapped reads, e.g. errors in sequence reads. The presence of SVs may also cause some reads to be unmappable. In the case of deletions, for example, when a read contains breakpoints of a deletion site, the read will contain two parts: one from the region prior to the deletion site and one from the region following the deletion site. The read may be unmappable because the read is a concatenation of the two parts and is not contained in the reference genome. The pairs with one read mapped and the other read unmapped are used in the split-read mapping methods. The mapped read in the pair is used as an anchor. The other read is split in the middle and then the two parts are attempted to map to the reference genome. If mapped correctly, the mapped split reads may reveal where the deletions occur. Recently there are more methods dedicated to find exact breakpoints. SRiC [11] is a split-read method mainly works on longer single reads like the Sanger and 454 reads. AGE [12] maps an assembled contig to a reference genome to detect the exact breakpoints of multiple SVs. There are also methods (e.g. CREST [13]) that do not detect the breakpoints themselves but rely on the exact breakpoints provided by mapping tools (through soft-clip mapping). A disadvantage of split-read mapping is that mapping split reads with a large gap is usually less efficient. Moreover, split reads may be mapped to wrong locations due to noises in the reads. Also, for the SVs with a breakpoint in a repetitive region, mapping may fail.

Despite there are increasing number of developed methods, calling structural variations from real sequence data remains a challenging computational problem. The challenges for calling structural variations with real sequence data include: (i) sequence data tends to be short and noisy (i.e. containing sequence errors or artifacts caused by errors in reads mapping), (ii) much current sequence data is at low coverage, and (iii) the volume of sequence data is often large. Therefore, much work is still needed to develop more accurate and efficient approaches for structural variation calling with low-coverage sequence data.

Recently, we have developed a computational approach for calling deletions from low-coverage sequence data [14]. This approach (implemented in the program SVseq1) integrates two existing deletion calling approaches (namely discordant insert size analysis and split-read mapping), and thus in principle it utilizes more information contained in the reads than the pure split-read mapping approaches. Since sequence data tends to be noisy, it is important to utilize more information contained in the data when calling deletions. Briefly, SVseq1 first tries to split a read (that cannot be mapped as a whole) and maps the prefix and suffix parts in two regions. The gap between the two mapped regions of the split read may correspond to a deletion. Since there may be more than one way of splitting for some reads and some mapped split reads may only be artifacts of sequence and/or mapping errors, we filter the *candidate *deletions (from the split-read mapping) using discordant insert size analysis. That is, we call a candidate deletion a true deletion only when the candidate deletions are supported by the discordant insert size analysis. Simulation results in [14] show that our method outperforms an existing method [10].

Our work in [14] makes progress toward improving deletion calling from sequence data. However, we notice that it has several disadvantages. The most severe issue is that it is difficult to determine the best way for splitting reads: due to noise in reads, there may be many equally good ways for splitting the reads. This not only leads to longer running time (due to the need to examine more candidate deletions), but also may introduce false positives. Moreover, split-read mapping tends to be slow especially for genome-scale data. At last, only deletion calling is supported in [14] and obviously other types of structural variations (e.g. insertions) may also be of interests to many downstream applications.

In this paper, we present our recent work that improves upon SVseq1 [14]. Our new approach is implemented in the program *SVseq2*. The following lists the main features of SVseq2.

1. Like SVseq1, SVseq2 calls deletions (and insertions) with exact breakpoints.

2. SVseq2 achieves more accurate calling through split-read mapping on focal regions. SVseq2 also has a much desired feature: there is no need to specify the maximum deletion size, which is often needed by other methods (e.g. [10,14]). SVseq2 is also much faster because it only needs to examine a small number of ways of splitting the reads.

3. SVseq2 utilizes new features of sequence reads mapping tools. Latest sequence reads mapping (e.g. BWA [3]) provides partial reads mapping (called soft-clips in BWA). These partially mapped reads are often provided in the sequence data. SVseq2 relies on the soft-clip mapping provided by reads mapping tools in part of the split-read mapping. This makes SVseq2 faster than SVseq1 and some other similar deletion finding programs (e.g. [10]).

4. SVseq2 is also easier to use: it only needs mapped sequence data (stored in a BAM file) and reference genome (stored in FASTA format) as input.

5. SVseq2 supports insertion calling from low-coverage sequence data.

## Methods

SVseq2 is mainly designed to reduce the number of falsely mapped split reads. In our previous method SVseq1 [14], split-read mapping is performed on a genomic region whose length depends on the maximum size of deletions to detect. Then, each mapped split read introduces a candidate deletion, which is then filtered through discordant pair analysis. Suppose one wants to find deletions up to 1 Mb long, SVseq1 needs to search for a region roughly 1 Mb long on the reference genome. Due to errors in reads and repeats in the genome, there may be many "hits" when split reads are mapped. Many falsely mapped splits reads are filtered with discordant pairs, but some may happen to pass the filtering step. Also, when the number of hits is large, it can be slow in finding all the hits and evaluating them. SVseq2 takes a different approach in calling deletions:

1. The mapped segment of a split read (from soft-clip mapping) is used as the starting point of split-read mapping. This utilizes new features of read mapping tool and speeds up the computation.

2. To locate the soft-clipped segment of the split read, we infer a focal region (i.e. the region that highly likely where the soft-clipped segment may be mapped) using the discordant read analysis. We will explain in the following how this step is performed.

3. The focal region is usually much shorter and thus there is less chance to introduce false positives. We then search for the occurrence of the second segment within the focal region using a semi-global alignment algorithm.

For insertions, SVseq2 also uses soft-clip mapping in locating the likely insertions. We now give a more detailed description on how SVseq2 calls deletions and insertions.

### Deletion calling

SVseq2 relies on two types of patterns formed by split reads to detect deletions.

**Figure 1:**
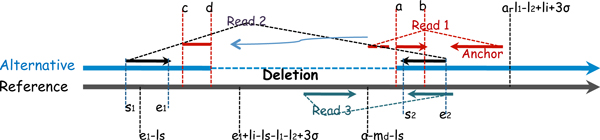
**Type I pattern of deletion calling**. Read 1 is a split read. Read 2 is a spanning pair. Read 3 is a pair on the other haplotype without the deletion.

**Figure 2:**
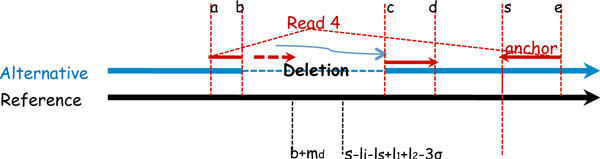
**Type II pattern of deletion calling**. Read 4 itself is a spanning pair. The left end is split.

• Type I pattern: the segment facing the anchor end is mapped (e.g. Read 1 in Figure 1).

• Type II pattern: the segment away from the anchor is mapped (e.g. Read 4 in Figure 2).

For type I pattern, the mapped segment of a split read based on soft-clip mapping faces the anchor. We denote the mapped location of the mapped segment as [*a, b*] (where *a *<*b*). To discover a deletion, the soft-clipped segment needs to be mapped to some region [*c, d*] (where *c *<*d *<*a*). We denote the length of the soft-clipped segment as *l_s _*= *d *- *c *+ 1. Because the length of the true deletion is not known, some existing split-read mapping methods (e.g. [10,14]) have a parameter on the maximum distance to search for the second (i.e. the soft-clipped) segment. Instead of searching in a large region, SVseq2 only searches a *focal *region by the guidance of spanning pairs. Our goal here is to infer where the soft-clipped segment is likely to start (i.e. the likely range of *c*). Our first observation is: even with low-coverage sequence data, a deletion is still likely to have at least one paired-end read whose two ends are located on different sides of the deletion (i.e. a spanning pair). Suppose there is a read pair whose two ends are mapped to [*s*_1 _, *e*_1 _] and [*s*_2 _, *e*_2 _] respectively on the reference genome (where *s*_1 _<*e*_1 _<*s*_2 _<*e*_2 _), and this pair is a spanning pair for the deletion, whose location is determined by the mapping of the soft-clipped segment of the split read. We let *l_i _*be the expected insert size and let *σ *be the standard deviation of the insert size. Note that *l_i _*measures the outer distance of the pair (i.e. the distance of the two farthest points of the two two reads). We denote the length of the two reads of the spanning pair as *l*_1 _and *l*_2 _respectively. Suppose the minimum deletion size to be detected by SVseq2 is *m_d_*. SVseq2 sets *m_d _*to be 50.

We first show where to find spanning pairs for a given split read.

**Lemma 1 ***For type-I pattern, s*_2 _≥ *a, and with high probability, we have s*_2 _≤ *a - l*_1 _- *l*_2 _+ *l_i _*+ 3*σ*.

**Proof 1 ***If s*_2 _*< a, then a is not a breakpoint. This does not agree with our underlying assumption that the mapped segment *[*a, b*] *corresponds to a deletion*.

*To give an upper bound on s*_2_, *note that a is the position of the right breakpoint. The rightmost position of e_1 _on the reference is a - l_del_, where l_del _is the length of the deletion. Now since with high probability, the distance between s*_2 _*and e_1 _is at most l_i _- l*_1 _- *l*_2 _*+ *3*σ + l_del _on the reference. So with high probability s*_2 _≤ (*a - l_del_*)*+ *(*l_i _- l*_1 _- *l*_2 _*+ *3*σ + l_del_*) *= a - l*_1 _- *l*_2 _*+ l_i _+ *3*σ*.

Lemma 1 states where the spanning pairs are very likely to be located. For a given split read, SVseq2 searches for reads mapped on the reverse strand within this region for spanning pairs.

Now suppose we find one spanning pair for the given split read. Recall the spanning pair is mapped to [*s*_1_, *e*_1 _] and [*s*_2_, *e*_2_]. The following lemma specifies the range of *c *(i.e. the starting point of the soft-clipped segment).

**Lemma 2 ***For type-I pattern, e*_1 _- *l_s _≤ c ≤ a - m_d _- l_s _*. *Moreover, with high probability, we have c ≤ e*_1 _*+ l_i _- l_s _- l*_1 _- *l*_2 _*+ *3*σ*.

**Proof 2 ***Note that the rightmost position a deletion can end is md bases to the left of a on the reference, because the minimum deletion size is m_d_. So c ≤ a - m_d_ - l_s_. Since the spanning pair *([*s*_1_, *e*_1_], [*s*_2_, *e*_2_]) *spans the deletion, we know the deletion must occur to the right of *[*s*_1_, *e*_1_]. *The leftmost position of the deletion is thus at least e1. Since the length of l_s _is to be mapped (to the left) from the left end of the deletion, we have c + l_s _≥ e*_1_.

*We now estimate how large c can be. Note that on the alternative chromosome (the chromosome with the deletion), the left and right breakpoints of the deletion become the same, and the left breakpoint of the deletion must be to the left of the starting position of the right end of the spanning pair. Thus, on the reference chromosome, with high probability, the left breakpoint is no bigger than e*_1 _*+ l_i _- l*_1 _- *l*_2 _*+ *3*σ*.

Lemma 2 states that we only need to search for the second segment of the split read within the region [*e*_1 _- *l_s_, min*(*e*_1 _+ *l_i _*- *l_s _*- *l*_1 _- *l*_2 _+ 3*σ, a *- *m_d _*- *l_s _*)]. This region is called the "*focal*" region for the split read being mapped and a spanning pair. In most current sequence data, the focal region is relatively small. For example, suppose *l_s _*= 50 (taken from a read of 100 bps long), *l*_1 _= *l*_2 _= 100, *l_i _*= 200 and *σ *= 50. Then the width of the focal region is not larger than 200. This is much smaller than the focal region that the original split-read mapping would have searched (which can be as long as 1 Mbps). Also, from Lemma 1, the width of the region for spanning pairs is at most 150.

The processing of split reads with type II pattern is similar in many aspects to that of type I pattern. A main difference between type I and type II patterns is that type II pattern does not need additional spanning pairs because the paired-end read itself is a spanning pair. This imposes an additional constraint on the focal region. Suppose the mapped segment of the split read is located at [*a, b*] and the mapped anchor is located at [*s, e*] (where *a *<*b *<*s *<*e *as shown in Figure 2). We let [*c, d*] be the location of the soft-clipped segment of the split read on the reference (where *b *<*c *<*d*). We let *l_s _*= *d *- *c *+ 1 be the length of the soft-clipped segment. We let *l*_1 _and *l*_2 _be the length of the two reads (i.e. *l*_2 _= *e *- *s *+ 1). *m_d_, l_i _*and *σ *are defined as before. The following lemma specifies where the soft-clipped segment is allowed to map.

Lemma 3 *b + m_d _≤ c ≤ s. Also, with high probability, we have s - l_i _- l_s _+ l*_1 _*+ l*_2 _- 3*σ ≤ c ≤ s*.

**Proof 3 ***Note that the leftmost position a deletion can start is m_d_ bases to the right of b on the reference, because the minimum deletion size is m_d _. Also, the breakpoint cannot go to the right side of s for there is no split on the anchor*.

*Note that the position of soft-clipped segment is constrained by the anchor position and the insert size. So the second inequality follows the same reasoning as in Lemma 2*.

Lemma 3 states that we only need to search for the second segment of Type II pattern of the split read within the focal region [*max*(*b *+ *m_d_, s *- *l_i _*- *l_s _*+ *l*_1 _+ *l*_2 _- 3*σ*), *s*]. For the cases when the split read is on the reverse strand, the method applied on them is essentially the same as when they are on the forward strand.

Our experience indicates that type I pattern is usually more reliable then type II pattern, because less errors are expected at the head of Illumina reads. Thus SVseq2 gives type I pattern higher weights than type II pattern when calling deletions. The weight of type I pattern is set to 3, and the weight is set to 1 for type II pattern. A cutoff value on the total weight (i.e. the sum of weights of supporting reads for a deletion) is used by SVseq2. The default cutoff value is set to 3, i.e. at least one type I pattern read is required or at least three type II pattern reads are required when there is no type I pattern read.

**Figure 3:**
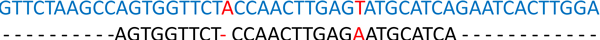
**SVseq2 uses a semi-global alignment algorithm**. The gaps "GTTCTAAGCC" and "GAATCACTTGGA" are without penalty. The gap at "TAC" and "T-C" are with penalty 3. The T-A mismatch are scored -1. Other Matches are scored 1 each. Total score is 23.

To search for the occurrence of a soft-clipped segment within a inferred focal region, SVseq2 uses a semi-global alignment algorithm, as illustrated in Figure 3. Briefly, we want to map the entire soft-clipped segment within the focal region. Thus, the gaps outside of the aligned positions for the focal region are without penalty, while we set the gap penalty within the read to 3. The similarity score is 1 for matches and -1 for mismatches. Since the focal region and the read are relatively short (e.g. several hundreds at most for the focal region and less than one hundred for Illumina reads), split-read mapping with sequence alignment can be performed relatively fast.

**Figure 4:**
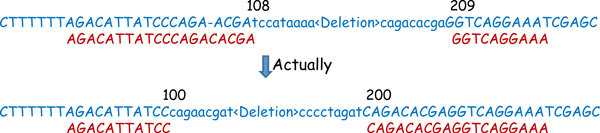
**Adjustment of breakpoints**. Length of a deletion can be adjusted. In the upper case, the left segment is mapped by Smith-Waterman algorithm with a gap and the length of the deletion is 101. In the lower case, the gap is removed and the length of the deletion is adjusted to 100.

Since the above split-read mapping method starts from soft-clip mapping, its accuracy depends on how accurate the soft-clip mapping is. Because soft-clip mapping is found through local sequence alignment, sometimes errors can be introduced by soft-clip mapping. We observe that one possible error in soft-clip mapping occurs when there is a gap in the soft-clip mapping. As shown in Figure 4, soft-clip mapping may align a segment longer than that in the true split read by introducing a false gap, while the true alignment can be achieved without gap by mapping a longer soft-clipped segment to a later position. When this occurs, the length of the detected deletion can be different from the real length. SVseq2 addresses this potential problem by using an adjustment step, which tries to find an optimized mapping of the entire read by avoiding errors (i.e. as shown in Figure 4). During the adjustment step, we examine all supporting split reads for some deletion. If each of the split reads can be adjusted to achieve a better mapping (i.e. by rearranging the split-read alignment as shown in Figure 4), then SVseq2 removes the gaps within these reads and adjusts the length of the deletion accordingly. If the reads do not agree with each other in terms of splitting positions, then SVseq2 takes a voting scheme by choosing split reads with higher alignment scores.

In practice, there may be more than one spanning pairs for a candidate deletion (corresponding to a split read). When the deletion is heterozygous in a diploid genome, Some spanning pairs may originate from the copy without the deletion while others from the copy with the deletion. Some other spanning pairs may be due to mapping errors. One possible scheme is to find a "consensus" focal region by combining information provided by multiple spanning pairs. SVseq2 simply takes the union of all the focal regions from all the possible spanning pairs. This is because there could be mapping errors in the spanning pairs, and thus SVseq2 takes a conservative estimate of the focal region. Our experience shows that the overall focal region is still relatively small and searching for split read can be performed relatively efficiently.

### Insertion finding

**Figure 5:**
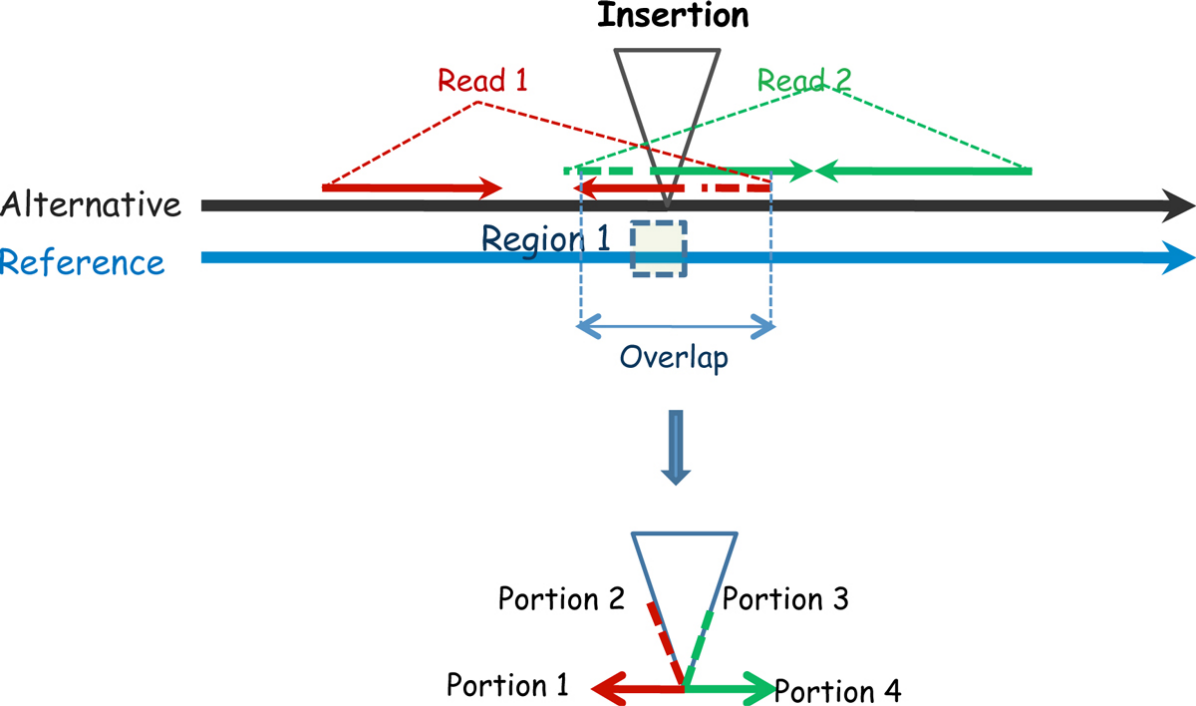
**Type III pattern in finding insertion**. Tail segments (portions 1 and 4) are mapped next to each other on the reference. The sequences of head segments (portions 2 and 3) belong to the inserted sequence.

SVseq2 uses the reads with head segments mapped with low quality (contains too many gaps or is soft-clipped) to detect insertions. (Here a head segment means the 5' portion of a read.) In particular, SVseq2 uses type III pattern: two mapped segments of split reads overlap but the two whole reads cannot be aligned well (see Figure 5). Both reads are from properly mapped pairs, and both have low quality mapping or soft-clip at the head segment. We consider a split read that has its tail mapped on the reverse strand. If another split read is from the other direction of the insertion, its split is very likely to be only located in a small region near the known breakpoint. For example, in Figure 5, knowing that read 1 is mapped with a possible breakpoint, then only the reads that have split in the short Region 1 have to be examined. As shown in Figure 5, if there is an insertion, then the heads of the reads are not from the reference genome. Thus, the overlapped portions of the two reads are unlikely to be aligned well. On the other hand, if there is no insertion, then the overlapped portions come from the same genomic region and should be aligned well. Because the not well mapped segments are from the heads of the Illumina reads, less errors are expected in these segments and their alignment is more reliable.

SVseq2 relies on pair wise sequence algorithm to align two overlapped reads. The parameters are the same as for the deletion case. If the score of the mapping over the length of the overlap is less than 0.1 then the pair is treated as evidence of a possible insertion. The default cutoff value of reads supporting an insertion for SVseq2 is 3. That is, at least another read in this region has the same split and passes the alignment test with the read in this pair on the different strand.

## Results and discussion

We apply SVseq2 on both simulated datasets and real datasets, comparing with SVseq1 [14] and Pindel 0.2.4d [10] on accuracy and efficiency. For deletion finding, the three methods are run on simulated population data, real individual and pooled data. For insertion finding, simulated individual data is used. The real sequence datasets (20101123 Illumina data) consist of the alignment files of 18 individuals on chromosome 20. Nine of the individuals are from the CEU population and the others are from the YRI population. These alignment datasets are mapped using BWA with soft-clips on NCBI human genome 37. The accuracy is evaluated according to the results by the 1000 Genomes Project ftp://ftp-trace.ncbi.nih.gov/1000genomes/ftp/technical/working/20110719_merged_sv_calls/. The results contain assembled deletions and the ones found by five SV detection tools of more than 1000 individuals (which include the ones used in this paper). The methods include BreakDancerMax1.1 [5], CNVnator [9], GenomeStrip v1.04 [15], EMBL/Delly and Pindel [10]. Since not all of the methods are able to provide exact breakpoints of deletions, evaluation of accuracy of methods is based on both a strict criterion and a less strict criterion. A called deletion is viewed correct by the strict one, if the length of the called deletion is the same as a deletion in the results by the benchmark. The less strict one only requires that a called deletion overlaps with a deletion in the benchmark, and at least 50% of the bases of the called deletion are supported. 

### Finding deletions using simulated pooled data

The simulated datasets with read length 100 from [14] are used in this paper to compare SVseq2 to SVseq1 and Pindel in terms of accuracy and sensitivity. The datasets are simulated from the sequence of chromosome 15 (100,338,915 bps in length) of NCBI human genome 36. The results of the copy number variation release paper of the 1000 Genomes Project [16] are based on this version of genome. The deletions of the 45 individuals from the CEU population reported by [16] are introduced to the simulation datasets (union.2010_06.deletions.genotypes.vcf.gz). Since the haplotypes of the deletions are not inferred in the file, for the heterogeneous deletions we arbitrarily place one such deletion to one of the two haplotypes of an individual. Since the deletions are usually far apart from each other, this may not have big effects on the accuracy of the simulation. A tool called wgsim https://github.com/lh3/wgsim is used with the "-h" option to generate paired-end reads from the two copies of genomes of an individual. Single nucleotide polymorphisms and small indels on each genome are simulated using the default parameters. All the datasets are generated with base error rate 2%. Paired-end reads are simulated with read length 100 and "outer distance" 500. Three datasets with coverage 3.2×, 4.2× and 6.4× are used. BWA, which provides soft-clips, is used with default parameters to map these simulated paired-end reads to the entire NCBI human genome 36.

The performance of finding deletions is compared among SVseq2, SVseq1 and Pindel, on these pooled datasets. The results are shown in Table 1. We can see that SVseq2 usually has the highest accuracy and sensitivity. Pindel usually has a high accuracy but lower sensitivity comparing with the other two methods. Mapping a soft-clipped segment of a split read to a focal region reduces the chance that this segment is mapped to wrong positions. Since the mapping approach of SVseq2 is more accurate, it does not need a higher cutoff to call deletions (recall that the cutoff value of 3 means that only one type I pattern read is needed). We can see that when coverage is higher (6.4×), the sensitivity of SVseq2 and SVseq1 is similar. But when coverage is lower (3.2×), the sensitivity of SVseq2 is higher than SVseq1. When the coverage or the frequency of a deletion is very low, SVseq2 may have a better chance of detecting it than using the other two methods.

**Table 1:** Comparison of SVseq2, SVseq1 and Pindel in simulation.

Coverage	Tool	Findings	True Positive	Accuracy (%)	Sensitivity (%)
3.2×	SVseq2	114	112	98	85
	SVseq1	111	108	97	82
	Pindel	91	90	99	68

4.2×	SVseq2	113	112	99	85
	SVseq1	117	109	93	83
	Pindel	91	90	99	68

6.4×	SVseq2	123	120	98	91
	SVseq1	128	120	94	91
	Pindel	103	102	99	77

Reads of length 100 on chromosome 15 with 132 deletions are simulated. The cutoff value of SVseq2 is 3. The cutoff value is 3 for SVseq1 and Pindel. Number of findings and true positives in each setting are reported.

**Figure 6:**
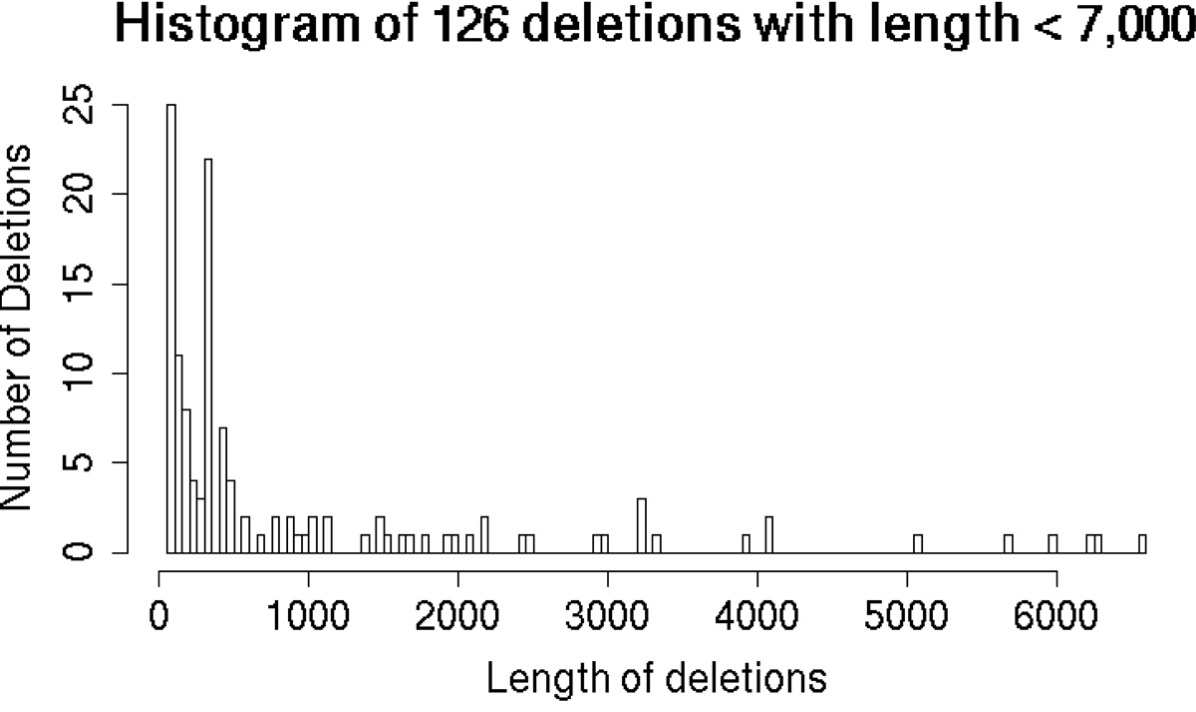
**Lengths of the deletions used in the simulation**. Histogram of the lengths of 126 deletions in the simulation with length less than 7, 000. There are other 6 deletions with length range from 7, 688 to 160, 798.

A called deletion is viewed correct in the comparison in Table 1 if the length of a called deletion is the same as the simulated length. The split-read approaches have the advantage of high resolution of breakpoints, while different approaches such as read depth and read pair methods are not suitable to find the exact breakpoints. The 132 deletions introduced into the simulation are only from 45 individuals of the CEU population on one chromosome, but the frequencies of the lengths of these deletions (see Figure 6) show the same trend with the frequencies of the deletions found by the 1000 Genomes Project (refer to Figure 2a of [16]). SVseq2 is able to detect both smaller and larger deletions. For example, in the 6.4× coverage setting, SVseq2 finds all the 6 larger deletions with length > 7, 000 and Pindel misses one deletion. For the 33 deletions with length in range 1, 000 to 7, 000, SVseq2 finds 29 and Pindel finds 25 deletions. For the 93 smaller deletions with length < 1, 000, the numbers are 85 and 72, respectively. The effectiveness of the SV finding methods may also be affected by sequence coverage. For example, CNVnator [9] is a read depth method that is very accurate on the 1000 Genomes Project's trio data. The resolution of breakpoints is also high when it is applied on high coverage data. But it does not perform as well on the low-coverage datasets, e.g. when it is applied on the 6.4× coverage data using bin size of 30, 100, 500 and 1, 000, CNVnator reports 510, 120, 74 and 53 deletions, with 44, 35, 14 and 8 correct (if the region of a reported deletion overlaps with the true region) respectively.

### Finding deletions using real individual data

The sequence data of five individuals from YRI population used by the 1000 Genomes Project is used to compare SVseq2, SVseq1 and Pindel on real individual data. The number of findings and true positives are shown in Table 2.

**Table 2:** Comparison of SVseq2, SVseq1 and Pindel using real individual data.

	SVseq2(3)			SVseq2(4)			SVseq1			Pindel		
	F	SE	SO	F	SE	SO	F	SE	SO	F	SE	SO
NA19311	48	38(79%)	44(92%)	24	24(100%)	24(100%)	15	14(93%)	14(93%)	3	2(67%)	2(67%)
NA19312	47	27(57%)	32(6872%)	19	16(84%)	16(84%)	28	12(43%)	20(72%)	9	2(22%)	7(78%)
NA19313	70	23(33%)	43(61%)	27	16(59%)	22(81%)	70	21(30%)	51(73%)	14	3(21%)	12(85%)
NA19316	17	14(82%)	16(94%)	4	3(75%)	4(100%)	3	2(67%)	3(100%)	7	5(71%)	6(86%)
NA19317	28	18(64%)	22(79%)	13	10(77%)	10(77%)	6	4(67%)	4(67%)	4	2(50%)	4(100%)

SVseq2 is run with cutoff values 3 and 4. The cutoff value is 3 for SVseq1 and Pindel. F stands for "Findings", SE for "Supported by exact breakpoints" and SO for "Supported by overlap". Numbers in the parenthesis are accuracies.

Using individual sequence data, SVseq2 is able to utilize split reads to call more deletions than SVseq1 and Pindel even when the coverage is very low. With cutoff value 3, SVseq2 finds the largest number of deletions and a large portion has supports by the benchmark. If a higher cutoff value 4 is used, most of the called deletions are supported by the benchmark. The number of findings is still larger than SVseq1 and Pindel, when using cutoff value 4.

### Finding deletions using real pooled data

Sequence reads from 9 individuals from CEU population are pooled together, and reads from 9 individuals from YRI populations are pooled together. SVseq2, SVseq1 and Pindel are tested using these two pooled datasets. Results are shown in Table 3.

**Table 3:** Comparison of SVseq2, Svseq1 and Pindel using real pooled data.

	SVseq2(3)			SVseq2(4)			SVseq1			Pindel		
	F	SE	SO	F	SE	SO	F	SE	SO	F	SE	SO
CEU	108	43(40%)	72(67%)	39	26(67%)	36(92%)	258	48(19%)	99(38%)	30	19(63%)	25(83%)

YRI	195	77(39%)	133(68%)	84	55(65%)	68(81%)	131	54(41%)	101(77%)	112	38(34%)	77(69%)

SVseq2 is run with cutoff values 3 and 4. The cutoff value is 3 for SVseq1 and Pindel. F stands for "Findings", SE for "Supported by exact breakpoints" and SO for "Supported by overlap". Numbers in the parenthesis are accuracies.

Using pooled data, all three methods are able to find more deletions than using individual data. SVseq2 finds more deletions using cutoff value 3 but false positive rate is increased too. Quite a portion of deletions found by SVseq2 using cutoff value 3 are missed by using cutoff value 4. Even pooling nine individuals together, many less frequent deletions still belong to single individuals. Because the sequence coverage is low, only one split read with soft-clipped mapping covers such a deletion (recall that the cutoff value 3 means one type I read). The quality of soft-clipped mapping provided by the mapping tools matters in finding SVs. If a mapping tool fails to perform soft-clip mapping on a split read, then this read is not used by SVseq2. By pooling more data from more individuals, more deletions are likely to be found by SVseq2.

### Simulation results for insertion

There are fewer insertion finding methods than deletion finding. Also, fewer insertions have been called and released than deletions. To simulate insertion, the release (CEU.trio.2010_06.novelsequences.sites.vcf) of the NA12878 individual is used in this paper. This individual has been sequenced at high coverage and the 1000 Genomes Project has released some inserted sequences of this individual. Chromosome 4 of NCBI human genome 36 is used in the simulation, since there are 13 (the highest number of) inserted sequences on this chromosome in the release for this individual. Each insertion is treated to be heterozygous and added into an arbitrary haplotype. Illumina reads with 20× coverage (so that each inserted sequence has 10× coverage) are simulated using wgism https://github.com/lh3/wgsim with insert size 230 and read length 100. The reads are mapped using BWA. Both SVseq2 and Pindel 0.2.4.d are tested to find insertions. SVseq2 finds 10 insertions with 9 true positives. Pindel finds 2 insertions, both of which are correct. One is found as "LI" and the other is found as "SI". The large insertion reported by Pindel as "LI" has 6 split reads supporting it, where 3 out of 6 are from the forward strand and the other 3 are from the reverse strand. Even at 20× coverage, split reads of type III pattern are not common in this simulation study. This simulation shows that SVseq2 is able to use fewer supporting reads to call insertions.

### Running time

Because the mapping of SVseq2 is performed on focal regions, the algorithm of SVseq2 is usually faster than SVseq1 and Pindel. The run time of SVseq2, SVseq1 and Pindel is compared in this paper on one dataset. The file (NA19312.chrom20.ILLUMINA.bwa.LWK.low_coverage.20101123.bam) from the 1000 Genomes Project is used. The chromosome is 63, 025, 520 bps in length and the file is about 5.4× coverage. Running time of the three methods with different settings is shown in Table 4. Each method is run using one thread on a 3192 MHz Intel Xeon workstation. It can be seen that SVseq2 is the fastest among the three methods in calling deletions. SVseq2 also runs faster in calling insertions than Pindel. Also note that the running time of SVseq1 and Pindel depends on the maximum event size. It can be seen that, if the maximum event size is set higher, both SVseq1 and Pindel will take even longer time to run. Before running SVseq1 and Pindel, running some additional scripts is needed to collect inputs for these two programs. Such preprocessing may take several minutes, which are not included in the table. SVseq2 takes the BAM file as input and there is no additional preprocessing.

**Table 4:** Comparison of SVseq2, SVseq1 and Pindel on running time.

Method	Max Event Size	SV Type	Time
SVseq2	None	DEL	1 m 48 s
	None	INS	20 s

SVseq1	35, 000	DEL	8 m 40 s
	1, 000, 000	DEL	82 m

Pindel	32, 368	DEL, INS, TD	7 m 40 s
	517, 888	DEL, INS, TD	93 m

DEL stands for deletion, INS for insertion and TD for Tandem repeat.

## Conclusions

There are four types of methods that use high throughput sequencing data to call SVs. Read depth methods and assembly methods usually need data with higher coverage. Read pair methods and read depth methods are not able to find exact breakpoints of SVs. Split-read mapping methods may find exact breakpoints of some SVs with low-coverage data. However, split-read mapping alone usually leads to significant false positives. Combining split-read mapping with other types of methods may increase the power in finding SVs. In this paper we describe an improved split-read mapping method to call SVs using low-coverage sequence data. We show that by using read pairs with discordant insert sizes, split-read mapping can be applied as mapping a segment of a split read on a focal region. Using the lemmas in the Methods section, we show that the length of the focal region can be much smaller than the maximum deletion size. Mapping split reads within a small focal region reduces the chance that a segment is aligned to incorrect positions. Thus, mapping split reads within focal regions leads to both higher accuracy and shorter running time. Applying on several datasets, we show that SVseq2 outperforms some other methods in both accuracy and efficiency. SVseq2 is more powerful compared to these methods when using very low coverage sequence data.

The split-read mapping approach in SVseq2 can still be improved, e.g. to better model the error patterns of high throughput sequencing data. For the situation when there are repeats in focal regions, insert size analysis might be helpful in finding correct mapping.

## Availability

The program SVseq2 can be downloaded at http://www.engr.uconn.edu/~jiz08001/.

## Competing interests

The authors declare that they have no competing interests.

## Authors' contributions

JZ designed algorithms, developed software, performed analysis and experiments, wrote the paper. JW contributed to performing analysis and experiments. YW designed the algorithms, wrote the paper and supervised the project. All authors have read and approved the final manuscript.
